# Supplementary figures and images for: Microgeographic differentiation in thermal and antipredator responses and their carry-over effects across life stages in a damselfly
Source: PLoS One. 2024 Feb 23;19(2):e0295707. doi: 10.1371/journal.pone.0295707 (PMC10889876; doi:10.1371/journal.pone.0295707)

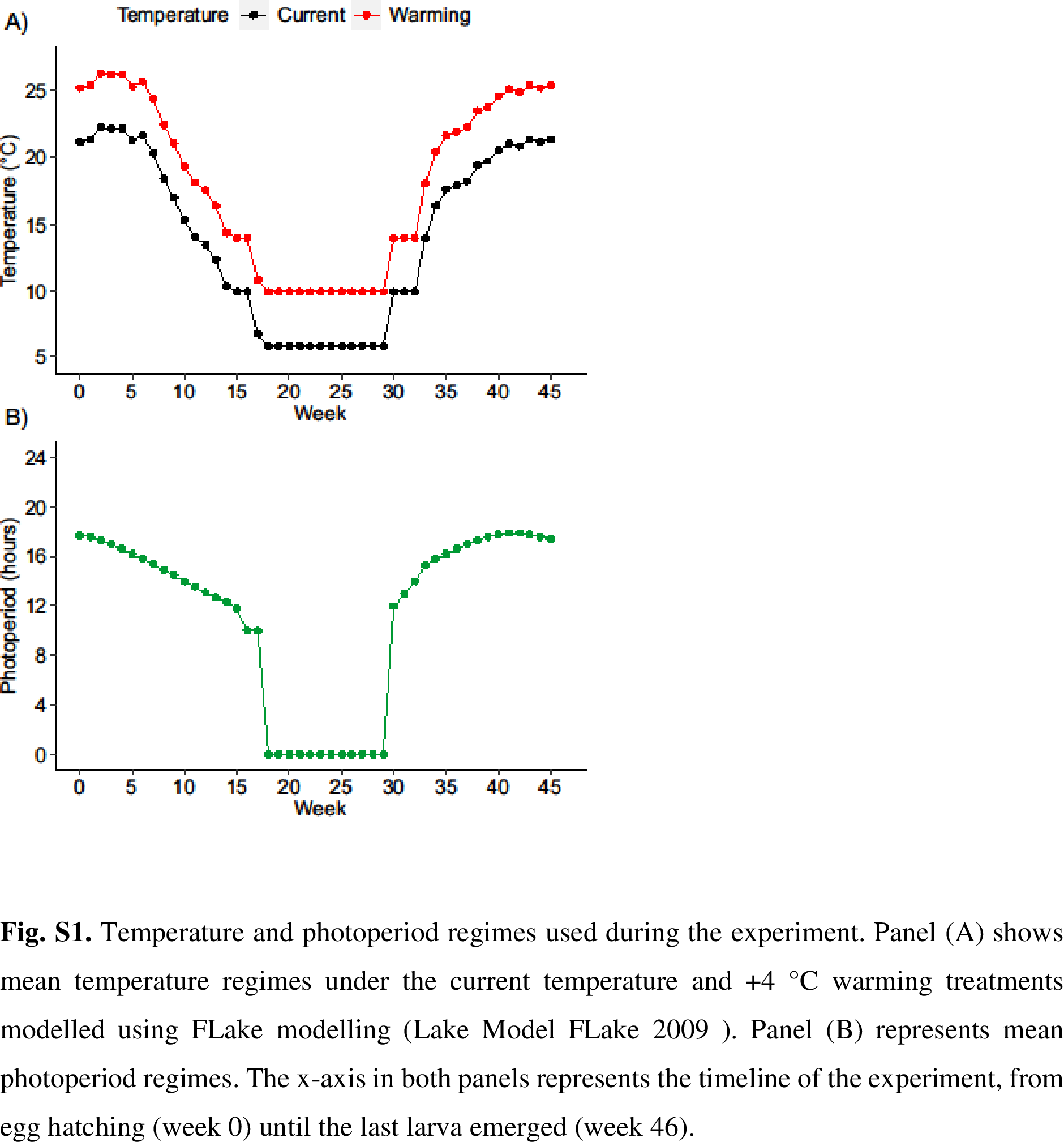

Supplement: S1 Fig — Panel (A) shows mean temperature regimes under the current temperature and +4°C warming treatments modeled using FLake modeling (Lake Model FLake. 2009). Panel (B) represents mean photoperiod regimes. The x-axis in both panels represents the timeline of the experiment, from egg hatching (week 0) until the last larva emerged (week 46). (TIF) [file pone.0295707.s001.tif]

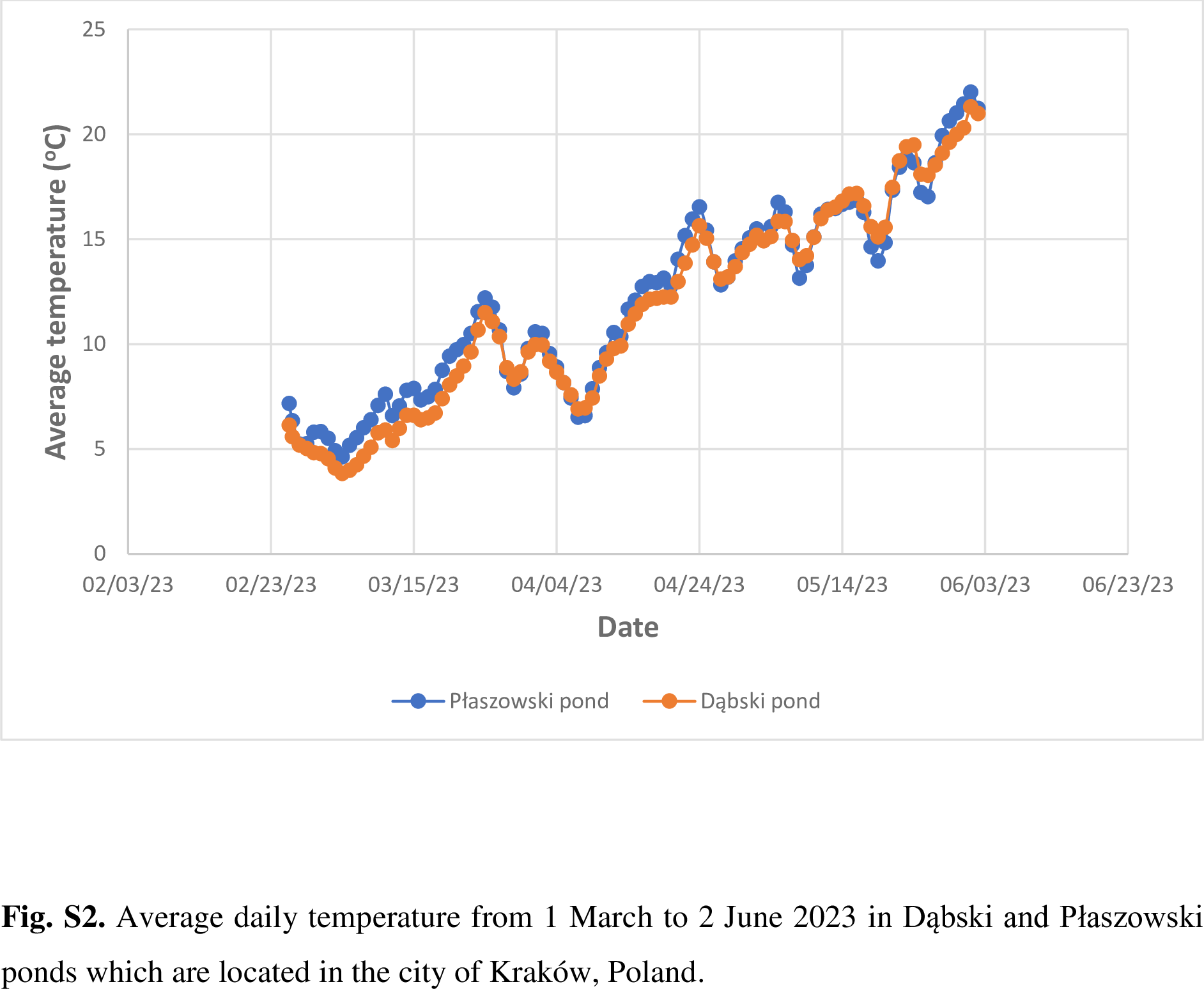

Supplement: S2 Fig — (TIF) [file pone.0295707.s002.tif]

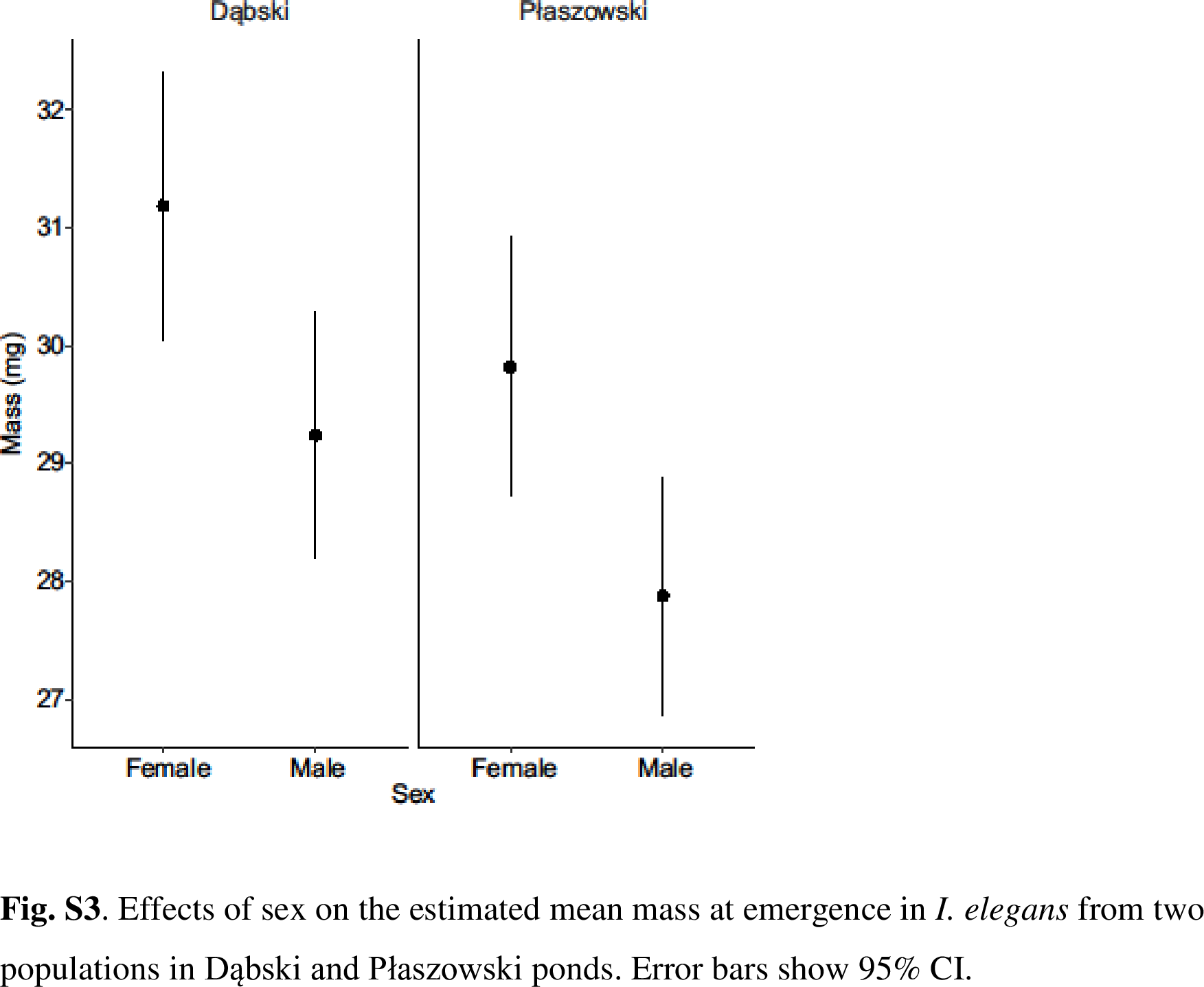

Supplement: S3 Fig — Error bars show 95% CI. (TIF) [file pone.0295707.s003.tif]
