# Supplementary material for: Microgeographic differentiation in thermal and antipredator responses and their carry-over effects across life stages in a damselfly
Source: PLoS One. 2024 Feb 23;19(2):e0295707. doi: 10.1371/journal.pone.0295707 (PMC10889876; doi:10.1371/journal.pone.0295707)
Supplement: S1 Table — In the experiment, the overwintering was shortened compared to nature. Therefore, during post-winter treatment, the dates for experimental values did not follow the current values in nature at a particular time point. (DOCX) [file pone.0295707.s004.docx]

**Table S1** The values of photoperiod, temperatures, and dates for which current values were taken. In the experiment, the overwintering was shortened compared to nature. Therefore, during post-winter treatment, the dates for experimental values did not follow the current values in nature at a particular time point.

| **Week, experimental** | **Date, nature** | **Date, experimental** | **Light hours** | **Dark hours** | **Temperature °C, current** | **Temperature °C, warming** |
| --- | --- | --- | --- | --- | --- | --- |
| 0 | 3 July | 3 July | 17:44 | 06:16 | 21.1 | 25.1 |
| 1 | 10 July | 10 July | 17:33 | 06:27 | 21.3 | 25.3 |
| 2 | 17 July | 17 July | 17:17 | 06:43 | 22.2 | 26.2 |
| 3 | 24 July | 24 July | 16:58 | 07:02 | 22.1 | 26.1 |
| 4 | 31 July | 31 July | 16:35 | 07:25 | 22.1 | 26.1 |
| 5 | 7 August | 7 August | 16:12 | 07:48 | 21.2 | 25.2 |
| 6 | 14 August | 14 August | 15:46 | 08:14 | 21.6 | 25.6 |
| 7 | 21 August | 21 August | 15:21 | 08:39 | 20.3 | 24.3 |
| 8 | 28 August | 28 August | 14:53 | 09:07 | 18.4 | 22.4 |
| 9 | 4 September | 4 September | 14:27 | 09:33 | 17.0 | 21.0 |
| 10 | 11 September | 11 September | 14:00 | 10:00 | 15.3 | 19.3 |
| 11 | 18 September | 18 September | 13:33 | 10:27 | 14.1 | 18.1 |
| 12 | 25 September | 25 September | 13:06 | 10:54 | 13.5 | 17.5 |
| 13 | 2 October | 2 October | 12:41 | 11:19 | 12.4 | 16.4 |
| 14 | 9 October | 9 October | 12:15 | 11:45 | 10.4 | 14.4 |
| 15 | 16 October | 16 October | 11:50 | 12:10 | 10 | 14 |
| 16 | 23 October | 23 October | 10:02 | 13:58 | 10 | 14 |
| 17 | 30 October | 30 October | 10:02 | 13:58 | 6.9 | 10.9 |
| 18 | 6 November | 6 November | 00:00 | 24:00 | 6 | 10 |
| 30 | 15 January | 2 April | 12:00 | 12:00 | 10 | 14 |
| 31 | 22 January | 9 April | 13:00 | 11:00 | 10 | 14 |
| 32 | 29 January | 16 April | 13:59 | 10:01 | 10 | 14 |
| 33 | 5 February | 23 April | 15:20 | 08:40 | 14 | 18 |
| 34 | 12 February | 30 April | 15:47 | 08:13 | 16.4 | 20.4 |
| 35 | 19 February | 7 May | 16:12 | 07:48 | 17.6 | 21.6 |
| 36 | 26 February | 14 May | 16:37 | 07:23 | 17.9 | 21.9 |
| 37 | 5 March | 21 May | 16:59 | 07:01 | 18.2 | 22.2 |
| 38 | 12 March | 28 May | 17:18 | 06:42 | 19.4 | 23.4 |
| 39 | 19 March | 4 June | 17:34 | 06:26 | 19.7 | 23.7 |
| 40 | 26 March | 11 June | 17:45 | 06:15 | 20.5 | 24.5 |
| 41 | 2 April | 18 June | 17:51 | 06:09 | 21.0 | 25.0 |
| 42 | 9 April | 25 June | 17:52 | 06:08 | 20.8 | 24.8 |
| 43 | 16 April | 2 July | 17:47 | 06:13 | 21.3 | 25.3 |
| 44 | 23 April | 9 July | 17:37 | 06:23 | 21.1 | 25.1 |
| 45 | 30 April | 16 July | 17:22 | 06:38 | 21.3 | 25.3 |
