# Supplementary material for: Microgeographic differentiation in thermal and antipredator responses and their carry-over effects across life stages in a damselfly
Source: PLoS One. 2024 Feb 23;19(2):e0295707. doi: 10.1371/journal.pone.0295707 (PMC10889876; doi:10.1371/journal.pone.0295707)
Supplement: S2 Table — Note that in cases when group sample size < 2, these groups were excluded from the analysis. (DOCX) [file pone.0295707.s005.docx]

**Table S2** Sample sizes across the experimental groups. Note that in cases when group sample size < 2, these groups were excluded from the analysis.

|  | **Dąbski pond** | | **Płaszowski pond** | |
| --- | --- | --- | --- | --- |
|  | Current | Warming | Current | Warming |
| Survival after 14 days  CC  PC  PP  SC  SS | 20/30  24/30  26/30  19/30  16/30 | 26/30  10/30  17/30  13/30  15/30 | 21/30  20/30  19/30  20/30  26/30 | 20/30  8/30  8/30  12/30  15/30 |
| Survival one day after emergence  CC  PC  PP  SC  SS | 2/30  2/27  19/29  6/24  4/28 | 15/29  7/30  10/30  1/30  5/29 | 1/28  11/30  10/29  11/29  14/30 | 6/24  3/27  4/30  7/30  7/30 |
| Mass at emergence  CC  PC  PP  SC  SS | 2  2  19  6  4 | 15  7  10  1  5 | 1  11  10  10  13 | 6  3  4  7  8 |
| Development time- Egg stage  C  P  S | 20  50  35 | 26  29  28 | 21  39  46 | 21  14  28 |
| Development time- larval stage  CC  PC  PP  SC  SS | 3  4  22  9  5 | 17  8  11  1  11 | 1  11  10  13  15 | 6  3  4  7  8 |
| Degree days- Egg stage  C  P  S | 20  50  35 | 26  29  28 | 21  39  46 | 21  14  28 |
| Degree days- larval stage  CC  PC  PP  SC  SS | 3  4  22  9  5 | 17  8  11  1  11 | 1  11  10  13  15 | 6  3  4  7  8 |
