# Supplementary material for: Microgeographic differentiation in thermal and antipredator responses and their carry-over effects across life stages in a damselfly
Source: PLoS One. 2024 Feb 23;19(2):e0295707. doi: 10.1371/journal.pone.0295707 (PMC10889876; doi:10.1371/journal.pone.0295707)
Supplement: S3 Table — (DOCX) [file pone.0295707.s006.docx]

**Table S3** Sample sizes of accidentally lost individuals across the experimental groups.

| **Predator group** | **Dąbski pond** | | **Płaszowski pond** | |
| --- | --- | --- | --- | --- |
|  | **Current**  **temp.** | **Warming**  **temp.** | **Current**  **temp.** | **Warming**  **temp.** |
| CC  PC  PP  SC  SS | 0  3  1  6  2 | 1  0  0  0  1 | 2  0  1  1  0 | 6  3  0  0  0 |
