# Supplementary material for: Microgeographic differentiation in thermal and antipredator responses and their carry-over effects across life stages in a damselfly
Source: PLoS One. 2024 Feb 23;19(2):e0295707. doi: 10.1371/journal.pone.0295707 (PMC10889876; doi:10.1371/journal.pone.0295707)
Supplement: S4 Table — Dąbski and Płaszowski ponds are located in the city of Kraków, Poland. Both ponds represent the same type of freshwater habitat, similar age, and the same original and current purpose. The distance between the ponds is 2,8 km, and both ponds are situated within the Wisła river valley. (DOCX) [file pone.0295707.s007.docx]

**Table S4** Study ponds characteristics. Dąbski and Płaszowski ponds are located in the city of Kraków, Poland. Both ponds represent the same type of freshwater habitat, similar age, and the same original and current purpose. The distance between the ponds is 2,8 km, and both ponds are situated within the Wisła river valley.

| **Abiotic and biotic characteristics** | **Dąbski pond** | **Płaszowski pond** |
| --- | --- | --- |
| Type of the pond | Anthropogenic | Anthropogenic |
| Historical purpose | Clay excavation | Clay excavation |
| Current purpose | Recreational | Recreational |
| Year since water filled | 1947 | 1930’s |
| Coordinates | 50°03'54.9"N 19°59'11.9"E | 50°02'33.4"N 19°58'01.8"E |
| Metres above mean sea level | 195 | 198 |
| Aquatic and semiaquatic vegetation | Submerged: *Ranunculus circinatus*, *Ceratophyllum demersum*.  Floating: *Nuphar lutea, Persicaria amphibia, Lemna minor, Lemna trisulca*.  Rushes: *Typha angustifolia, Alisma plantago-aquatica, Sparganium simplex*.  * Kurzyński J, Mielnicka B 2005 Staw Dąbski. Przyroda. [w] Skarby Przyrody i Kultury Krakowa i Okolic. | Submerged: *Ceratophyllum demersum*, *Batrachium aquatile, Najas marina, Utricularia vulgaris*.  Floating: *Persicaria amphibia, Persicaria hydropiper*, *Lemna minor*.  Rushes: *Equisetum telmateia,*  *Phragmites australis*, *Typha angustifolia, T. latifolia, Hottonia palustris, Juncus tenuis, Schoenoplectus lacustris*.  * Żurek R et al. 2017 Staw Płaszowski w Krakowie ‒ II. Biocenozy. Raport. |
| Size | 2,18 he | ca. 9 he |
| Depth and water level stability | Maximum ca. 5-6 m  Rather stable water level  * Sniegula S, personal observation. | Maximum 2,83 m, average 1,26 m  Recent periodic lowering of water surface  associated with excessively dense development in the vicinity of the pond, the draining of excavations for the construction of new buildings and atmospheric conditions, including summer draughts.  * <https://dziennikpolski24.pl/staw-plaszowski-eksperci-radza-by-dolac-wody-z-bagrow/ar/10068924>; access: 29.03.2019  * Sniegula S, personal observation |
| Vertical temperature gradient, range | Minor | Considerable, 28,2°C – 17,0°C measured at the pick of the growth season |
| Spring-fens at the bottom | Absent | Present, numerous (ca. 40 springs) |
| Species preferring well oxygenated water | Absent | *Chara hispida* and *Nitellopsis obtus* |
| Thermophilic species | Absent | *Marsilea quadrifolia*  * Bonk M 2014 Stwierdzenie marsylii czterolistnej *Marsylea quadrifolia* L. w Krakowie Wszechświat, 115(10), 12 |
| Fish composition | *Esox lucius, Rutilus rutilus, Tinca tinca, Carassius auratus gibelio, Rhadeus sericeus, Perca fluviatilis* | *Scardinius erythrophthalmus*, *Esox lucius, Tinca tinca, Carassius carassius, Silurus glanis, Perccottus glenii****,*** *Perca fluviatilis* |
| Crayfish composition | - Nobel crayfish *Astacus astacus*, last record in 2015 (Maciej Bonk, unpublished data);  - spiny-chick crayfish *Faxonius limosus* recorded from 2018 until current study;  - swamp crayfish *Procambarus clarkii* recorded from 2018 until current study  * Maciaszek R, Bonk M and Strużyński W 2019 New records of the invasive red swamp crayfish *Procambarus clarkii* (Girard, 1852) (Decapoda: Cambaridae) from Poland. Knowl. Manag. Aquat. Ecosyst. 2019, 420, 39.  * Stanek Ł, Wiehle D, Szybak F. 2015. Inwentaryzacja raków  występujących na terenie użytku ekologicznego Staw Dąbski  Kraków: Wydział Kształtowania Środowiska UMK, 3–21. | - Danube crayfish *Astacus leptodactylus*, last record in 2015.  * Maciej Bonk, unpublished data. |
| Water temperature | Average water temperature between 1 March 2023 – 2 June 2023 = 11.8 °C (see Fig. S2 and Table S5).  Logger reads between 25-31 August 2022 at the depth ca. 50 cm mean = 26 °C (min and max, 24.5 °C - 27.7 °C).  Comment: The average air temperature in August 2022 in Poland was 20.5°C and was as much as 2.0 degrees higher than the long-term average for that month (climatic normal period 1991-2020). This year's August should be included in the months of extremely warm thermally (Institute of Meteorology and Water Management - National Research Institute, <https://www.imgw.pl>). | Average water temperature between 1 March 2023 – 2 June 2023 = 12.4 °C (see Fig. S2 and Table S5).  Logger reads from 4 August 2017 (noon hours) at the depth 50-70 cm = 28.1- 28.2 °C.  * Baś G et al. 2017 *Wykonanie opracowania przyrodniczego obejmującego cały ekosystem Stawu Płaszowskiego* (Nr 141/2017). Kraków, wrzesień 2017. |
