# Supplementary material for: Microgeographic differentiation in thermal and antipredator responses and their carry-over effects across life stages in a damselfly
Source: PLoS One. 2024 Feb 23;19(2):e0295707. doi: 10.1371/journal.pone.0295707 (PMC10889876; doi:10.1371/journal.pone.0295707)
Supplement: S5 Table — Data were extracted from five loggers installed in the two ponds (three loggers in Płaszowski pond and two loggers in Dąbski pond) ca. 40 cm below the water surface. Significant p-values are in bold. (DOCX) [file pone.0295707.s008.docx]

**Table S5** Average daily temperature differentiation between Dąbski and Płaszowski ponds from 1 March to 2 June 2023. Data were extracted from five loggers installed in the two ponds (three loggers in Płaszowski pond and two loggers in Dąbski pond) ca. 40 cm below the water surface. Significant p-values are in bold.

| **Predictor** | **df** | **Chisq** | **p-value** |
| --- | --- | --- | --- |
| Average daily temperature  Pond  Day | 1  97 | 86.76  243.56 | **< 0.001**  < **0.001** |
