# Supplementary material for: Microgeographic differentiation in thermal and antipredator responses and their carry-over effects across life stages in a damselfly
Source: PLoS One. 2024 Feb 23;19(2):e0295707. doi: 10.1371/journal.pone.0295707 (PMC10889876; doi:10.1371/journal.pone.0295707)
Supplement: Attachment — Submitted filename: PONE-D-23-24816_reviewer 1.pdf [file pone.0295707.s009.pdf]

PLOS ONE

--Microgeographic differentiation in thermal and antipredator responses and their carry-over effects across life stages in a damselfly

--Manuscript Draft--

**Manuscript Number:**	PONE-D-23-24816
**Article Type:**	Research Article
**Full Title:**	Microgeographic differentiation in thermal and antipredator responses and their carry-over effects across life stages in a damselfly
**Short Title:**	Microgeographic differentiation in thermal and antipredator responses across life stages in a damselfly
**Corresponding Author:**	Nermen Amer<br>Cairo University<br>EGYPT
**Keywords:**	biological invasion, carry-over effect, non-consumptive predator effect, phenotypic plasticity, thermal adaptation, naïve prey hypothesis
**Abstract:**	Global warming and invasive species can impose a large impact on the condition of native species. However, we know relatively little about how these two factors, individually and in combination, shape phenotypes in ectotherms across life stages and how this can differ between populations. We investigated the non-consumptive predator effects (NCEs) imposed by native (perch) and invasive (signal crayfish) predators experienced only during the egg stage or during both the egg and larval stages in combination with warming on adult life history traits of the damselfly Ischnura elegans. To explore microgeographic differentiation, we compared two nearby populations differing in thermal conditions and predator history. Warming and predator cues from perch positively affected damselfly survival, possibly because the warmer temperature was closer to the the optimal temperature for survival and because of predator stress acclimation phenomena. While warming decreased, predator cues increased larval development time with a proportionally stronger effect of signal crayfish cues experienced during the egg stage, indicating a negative carry-over effect from egg to larva. Warming and predator cues increased mass at emergence, with the predator effect driven mainly by exposure to signal crayfish cues during the egg stage, indicating a positive carry-over effect from egg to adult. Notably, warming and predator effects were not consistent across the two studied populations, suggesting a phenotypic signal of adaptation at a microgeographic scale to thermal conditions and predator history. The results point out that population- and life-stage-specific responses in life-history traits to NCEs are needed to predict fitness consequences of exposure to native and invasive predators and warming in prey at a microgeographic scale.
**Order of Authors:**	Nermeen Amer
	Robby Stoks
	Andrzej Antoł
	Szymon Sniegula
**Additional Information:**	
**Question**	**Response**
**Financial Disclosure**<br><br>Enter a financial disclosure statement that describes the sources of funding for the work included in this submission. Review the submission guidelines for detailed requirements. View published research articles from *PLOS ONE* for specific	This research was supported by the National Science Center- Grant OPUS 17 (grant no. 2019/33/B/NZ8/00521). The funders had no role in study design, data collection, and analysis, decision to publish, or preparation of the manuscript.

Powered by Editorial Manager® and ProduXion Manager® from Aries Systems Corporation

examples.<br><br>This statement is required for submission and **will appear in the published article** if the submission is accepted. Please make sure it is accurate.<br><br>**Unfunded studies**<br>Enter: *The author(s) received no specific funding for this work.*<br><br>**Funded studies**<br>Enter a statement with the following details:<br>• Initials of the authors who received each award<br>• Grant numbers awarded to each author<br>• The full name of each funder<br>• URL of each funder website<br>• Did the sponsors or funders play any role in the study design, data collection and analysis, decision to publish, or preparation of the manuscript?<br>• **NO** - Include this sentence at the end of your statement: *The funders had no role in study design, data collection and analysis, decision to publish, or preparation of the manuscript.*<br>• **YES** - Specify the role(s) played.<br><br>* typeset	
**Competing Interests**<br><br>Use the instructions below to enter a competing interest statement for this submission. On behalf of all authors, disclose any competing interests that could be perceived to bias this work—acknowledging all financial support and any other relevant financial or non-financial competing interests.<br><br>This statement is required for submission and **will appear in the published article** if the submission is accepted. Please make sure it is accurate and that any funding sources listed in your Funding Information later in the submission form are also declared in your Financial Disclosure statement.	The authors have declared that no competing interests exist.

View published research articles from *PLOS ONE* for specific examples.<br><br>**NO authors have competing interests**<br><br>Enter: *The authors have declared that no competing interests exist.*<br><br>**Authors with competing interests**<br><br>Enter competing interest details beginning with this statement:<br><br>*I have read the journal's policy and the authors of this manuscript have the following competing interests: [insert competing interests here]*<br><br>* typeset	
**Ethics Statement**<br><br>Enter an ethics statement for this submission. This statement is required if the study involved:<br><br>• Human participants<br>• Human specimens or tissue<br>• Vertebrate animals or cephalopods<br>• Vertebrate embryos or tissues<br>• Field research<br><br>Write "N/A" if the submission does not require an ethics statement.<br><br>General guidance is provided below. Consult the submission guidelines for detailed instructions. **Make sure that all information entered here is included in the Methods section of the manuscript.**	N/A

**Format for specific study types**<br><br>**Human Subject Research (involving human participants and/or tissue)**<br>• Give the name of the institutional review board or ethics committee that approved the study<br>• Include the approval number and/or a statement indicating approval of this research<br>• Indicate the form of consent obtained (written/oral) or the reason that consent was not obtained (e.g. the data were analyzed anonymously)<br><br>**Animal Research (involving vertebrate animals, embryos or tissues)**<br>• Provide the name of the Institutional Animal Care and Use Committee (IACUC) or other relevant ethics board that reviewed the study protocol, and indicate whether they approved this research or granted a formal waiver of ethical approval<br>• Include an approval number if one was obtained<br>• If the study involved *non-human primates*, add *additional details* about animal welfare and steps taken to ameliorate suffering<br>• If anesthesia, euthanasia, or any kind of animal sacrifice is part of the study, include briefly which substances and/or methods were applied<br><br>**Field Research**<br><br>Include the following details if this study involves the collection of plant, animal, or other materials from a natural setting:<br>• Field permit number<br>• Name of the institution or relevant body that granted permission	
**Data Availability**<br><br>Authors are required to make all data underlying the findings described fully available, without restriction, and from the time of publication. PLOS allows rare exceptions to address legal and ethical concerns. See the PLOS Data Policy and FAQ for detailed information.	Yes - all data are fully available without restriction

A Data Availability Statement describing where the data can be found is required at submission. Your answers to this question constitute the Data Availability Statement and **will be published in the article**, if accepted.<br><br>**Important:** Stating 'data available on request from the author' is not sufficient. If your data are only available upon request, select 'No' for the first question and explain your exceptional situation in the text box.<br><br>Do the authors confirm that all data underlying the findings described in their manuscript are fully available without restriction?	
**Describe where the data may be found in full sentences. If you are copying our sample text, replace any instances of XXX with the appropriate details.**<br><br>• If the data are **held or will be held in a public repository**, include URLs, accession numbers or DOIs. If this information will only be available after acceptance, indicate this by ticking the box below. For example: *All XXX files are available from the XXX database (accession number(s) XXX, XXX.*).<br>• If the data are all contained **within the manuscript and/or Supporting Information files**, enter the following: *All relevant data are within the manuscript and its Supporting Information files.*<br>• If neither of these applies but you are able to provide **details of access elsewhere**, with or without limitations, please do so. For example:<br><br>*Data cannot be shared publicly because of [XXX]. Data are available from the XXX Institutional Data Access / Ethics Committee (contact via XXX) for researchers who meet the criteria for access to confidential data.*<br><br>*The data underlying the results presented in the study are available from (include the name of the third party*	N/A

and contact information or URL).<br>• This text is appropriate if the data are owned by a third party and authors do not have permission to share the data.<br><br>* typeset	
Additional data availability information:	Tick here if the URLs/accession numbers/DOIs will be available only after acceptance of the manuscript for publication so that we can ensure their inclusion before publication.; Tick here if your circumstances are not covered by the questions above and you need the journal’s help to make your data available.

Powered by Editorial Manager® and ProduXion Manager® from Aries Systems Corporation

Manuscript Click here to access/download;Manuscript;Manuscript.docx

Microgeographic differentiation in thermal and antipredator responses and their carry-over effects across life stages in a damselfly

Nermeen R. Amer[1,2], Robby Stoks[3], Andrzej Antoł[1], Szymon Sniegula[1]

[1]Institute of Nature Conservation, Polish Academy of Sciences, 31-120 Krakow, Poland

[2] Entomology Department, Faculty of Science, Cairo University, Giza 12613, Egypt

[3] Evolutionary Stress Ecology and Ecotoxicology, University of Leuven, 3000 Leuven, Belgium

NRA and SS contributed equally to the manuscript

Key words: biological invasion, carry-over effect, non-consumptive predator effect, phenotypic plasticity, thermal adaptation, naïve prey hypothesis

Abstract

~~Global warming and invasive species can impose a large impact on the condition of native species~~. However, we know relatively little about how these two factors, individually and in combination, shape phenotypes in ectotherms across life stages and how this can differ between populations. We investigated the non-consumptive predator effects (NCEs) imposed by native (perch) and invasive (signal crayfish) predators experienced only during the egg stage or during both the egg and larval stages in combination with warming on adult life history traits of the damselfly *Ischnura elegans*. To explore microgeographic differentiation, we compared two nearby populations differing in thermal conditions and predator history. Warming and predator cues from perch positively affected damselfly survival, possibly because the warmer temperature was closer to the the optimal temperature for survival and because of predator stress acclimation phenomena. While warming decreased, predator cues increased larval development time with a proportionally stronger effect of signal crayfish cues experienced during the egg stage, indicating a negative carry-over effect from egg to larva. Warming and predator cues increased mass at emergence, with the predator effect driven mainly by exposure to signal crayfish cues during the egg stage,

indicating a positive carry-over effect from egg to adult. Notably, warming and predator effects were not consistent across the two studied populations, suggesting a phenotypic signal of adaptation at a microgeographic scale to thermal conditions and predator history. The results point out that population- and life-stage-specific responses in life-history traits to NCEs are needed to predict fitness consequences of exposure to native and invasive predators and warming in prey at a microgeographic scale.

Introduction

As organisms are typically exposed to multiple stressors which may interact, it is important to study the individual and combined effects of these stressors to accurately assess their impact [1–4]. Two major stressors mediated by human activity, the introduction of invasive predators and warming can drastically affect native prey communities and whole ecosystems. For example, the introduction of an invasive alien predator might dramatically reduce native prey communities, as the later have not evolved recognition ability and hence defence mechanisms against new predators [5–7], supporting the so-called naïve prey hypothesis [5,8,9]. Warming may alter ectotherms' development rates, resulting in changed phenological events such as advanced hatching and emergence dates [10–12], which may, in turn, affect predator-prey interactions [8,13–16].

In many animals with a complex life cycle, i.e. which go through discrete egg, larva, and adult stages, the degree of predation risk and the antipredator responses may differ between life stages [17–19]. For instance, the presence of non-consumptive predator cues of *Perca fluviatilis* fish during the egg stage decreased survival, but only up to two weeks after larval hatching, while the same exposure during the larval stage decreased survival and increased larval development time [17]. Moreover, predation risk imposed during a particular ontogenetic stage can be carried over to the following stage(s). This was shown in different invertebrate groups, including insects

[20–22]. For example, exposure of freshwater shrimps to predation risk during the embryonic stage led to changes in larval morphology and development time [21]. Identifying and exploring carry-over effects is essential to evaluate the total impact of predation risk on prey performance.

Prey responses to predation risk and warming can differ between populations, even at a microgeographic scale [23–26]. It has been suggested that population-specific responses might be driven by habitat-specific exposure history to these potential stressors [24,27,28]. For example, thermal performance in odonate larvae differed considerably between urban and adjacent rural sites [23,29]. The mosquito larvae *Culex restuans* showed stronger antipredator responses to salamander chemical cues in a population with increased predator density [26]. The ability of organisms to survive and grow under global warming not only depends on the physiological adjustments [30] but also on the ability of organisms to deal with the changes resulting from the interaction between temperature and predation risk [31]. For example, anti-predatory responses may change under warming [31]. This asks for cross-population studies that combine the effects of predation and warming-related stress in order to predict organismal performance under human-induced global change.

Here, we tested whether predation risk imposed throughout the egg stage only (acute exposition) or throughout the egg and larval stages (chronic exposition) affected prey traits at adult emergence through carry-over effects, and to what extent this can be modulated by warming in a semiaquatic insect, the damselfly *Ischnura elegans*. We compared the responses to cues from a native predator, perch fish (*Perca fluviatilis*) versus those from an invasive alien predator, the signal crayfish (*Pacifastacus leniusculus*). Along with the naïve prey hypothesis [8], we expect that the damselfly will only respond to native predator cues with lower survival, delayed egg hatching and adult emergence, and decreased mass at emergence. We expect the predator-induced

carry-over effects to the adult stage to be higher under chronic exposure to the predator cues. Finally, we expect warming will increase development rate, decrease survival and mass at emergence, and lower the predator-induced carry-over effect to the adult stage since higher temperature shortens the exposure time to predator cues.

Material and methods

Study species and study ponds

I. elegans is a common damselfly occurring in Europe from northern Spain to central-southern Sweden [32]. In central Europe, the damselfly has one or two generations per year [33] with diapausing larvae as the overwintering stage [34]. Larvae hatch a couple of weeks after egg laying. Aquatic egg and larval stages commonly share habitats with top predators such as fish and crayfish [17,35–37]. It was shown that predator cues affect *I. elegans* damselfly life history and physiology during both the egg stage and larval stage [13,17,38].

The study animals were collected from two nearby ponds, Dąbski and Płaszowski, within the city of Kraków, southern Poland. Both ponds are situated within the Wisła river valley and are separated by a distance of 2.7 km. Based on the short distance, and previously published literature [39], we assume that gene flow between these two populations is high. The ponds differ considerably in size, depth, thermal conditions, history of native and invasive crayfish occurrence, and other biotic and abiotic characteristics, which are described in detail in Table S4. Based on pond characteristics and our temperature logger reads, Dąbski pond holds on average colder water than Płaszowski pond (Figure S2, Table S5). Dąbski pond was reported to obtain one native crayfish, the noble crayfish (*Astacus astacus*), and two invasive alien crayfish species, spiny-chick crayfish (*Faxonius limosus*) and swamp crayfish (*Procambarus clarkia*). Instead, Płaszowski pond contains only one native crayfish species, Danube crayfish (*Astacus leptodactylus*). We used cues

from two different predator species to induce non-consumptive predator effects (NCE). The first type of cues came from the native European perch (*P. fluviatilis*) which is a common fish species in Europe [40]. Both *I. elegans* sampling ponds hold perch populations (Table S4). The second type of predator cues came from the invasive alien signal crayfish (*P. leniusculus*) that does not co-occur with *I. elegans* in the sampling ponds (Table S4). Signal crayfish is native to North America. The crayfish was introduced to Europe, and Scandinavia specifically, in the 1960s. In the following years, the species was introduced to other European countries, including Poland. Currently, it is the most spread non-native crayfish on the continent and has a negative impact on local biodiversity [41]. The distance between the crayfish population and the damselfly collection ponds is ca. 200 km (Maciej Bonk and Rafał Maciaszek, pers. communication).

Collection and housing of I. elegans

Adult females *I. elegans* in copula were collected from the two ponds on 5 July 2020 using a standard method [42]. Females were placed in plastic containers with wet filter paper for egg laying. Containers with females were placed in a Styrofoam box with frozen cartridges to keep the temperature low. Females were transported by car to the Institute of Nature Conservation, Polish Academy of Sciences (INC PAS) in Kraków, where the laboratory experiment was run. Females were kept in a room with a natural photoperiod and a temperature of ca. 22 °C until egg laying. Newly laid clutches were transferred to two incubators (Pol-Eko ST700). The temperatures at which eggs and larvae were reared were adjusted once a week to follow seasonal changes of mean weekly temperatures in shallow water, which is the optimal habitat for damselfly larvae [34].

Experimental setup

We set up two temperature treatments (current temperature and +4 °C warming) crossed with three predator cue treatments in the egg stage and five predation cue treatments in the larval stage (Figure

1). At hatching, the egg control group continued as a larval control group (CC). The egg perch cue group was divided into larval control (PC) and larval perch cue (PP) groups. The egg signal crayfish cue group was divided into larval control (SC) and larval signal crayfish cue (SS) groups (Figure 1).

Figure 1. Experimental design. *Ischnura elegans* eggs were assigned to groups receiving no predator cues (control), native European perch cues (perch), or invasive alien signal crayfish cues (signal crayfish). At hatching, the egg control group continued as larval control group (CC). The egg perch cue group was divided into larval control (PC) and larval perch cue (PP) groups. The egg signal crayfish cue group was divided into larval control (SC) and larval signal crayfish cue (SS) groups.

The two temperature treatments were the current temperature treatment which mimicked the current temperature in the collection site and the warming treatment where the current temperature was increased by 4 °C to mimic the predicted temperature change by 2100 [43]. The current temperatures were extracted from the Lake Model Flake [44]; in previous studies [45,46] it was shown this gives a reliable estimate of the field-measured water temperatures in ponds inhabited by the study species [47]. Except for overwintering conditions (see below), we followed weekly seasonal changes of daylight (photoperiod), with the inclusion of the Civil twilight at the sampling latitude. During the simulated winter, the temperature was set constant at 6 °C in the current temperature treatment and at 10 °C in the warming treatment. The light during winter was switched off. For the details of the applied temperature and photoperiod regimes, we refer to Figure S1 and Table S1.

We installed three predator cue treatment groups in the egg stage: control, perch cues and signal crayfish cues. At hatching, these gave rise to five larval predator cue treatment groups (Figure 1).

The control egg group continued as a control group in the larval stage. The two other predator cue treatment groups during the egg stage were each divided into two groups: a group that continued experiencing the same predator cues during the larval stage and a control group that did no longer exposed to predator cues during the larval stage. The latter group was designed to test for carry-over predator effect from the egg stage through the larval to the adult stages. Perch and signal crayfish cues were never combined.

To start, every egg clutch (each from a single female) was divided into six parts that were placed in separate plastic containers (15 x 11 x 7.5 cm) filled with 600 ml of water. In these containers, all clutches were pooled per population (Dąbski and Płaszowski pond). Water consisted of ¾ dechlorinated tap water and ¼ of dechlorinated tap water that, depending on the predator cue treatment contained predator cues originating from perch or signal crayfish. Eggs from every female (i.e., family) were present in every treatment combination during the egg stage: three predation cues (perch, signal crayfish, and control) crossed with two temperature (current temperature and warming) treatments. Water refill took place every second day to keep the predator cue levels approximately constant [48]. At hatching, larvae were transferred to other containers (19 x 12 x 9 cm) filled with 1 l of water and kept in groups of 15 individuals for another 14 days. Group rearing during initial instars increases larval survival [49]. Next, larvae were individually placed in plastic cups (height 9 cm, diameter 4 cm), each filled with 100 ml of water. Water refill in cups holding individual larvae was analogous as in group-rearing containers (¾ dechlorinated tap water and ¼ of dechlorinated tap water with or without predator cues; refilled every second day). The larvae were fed twice a day (morning and afternoon) during weekdays and once a day during weekend days with laboratory-cultured *Artemia* sp. nauplii. For the first 14 days, larvae kept in groups received 10 ml of *Artemia* solution/container, while larvae kept in individual

cups received 1 ml of an *Artemia* solution that contained on average 198.5 (SD = 92.4) nauplii/ml (N = 38 samples counted). During the simulated winter, larvae were fed once a day throughout the week. When larvae reached the pre-final instar (F-1), each individual was additionally provided with one living chironomid larva of a standardized size three times a week (Monday, Wednesday, and Friday).

Collection and housing of the predators

The predators were collected a couple of weeks before we started the experiment. We collected perch from Dobczyce Lake in southern Poland (49°52′18.316″N, 20°2′30.937″E) and signal crayfish from Hańcza Lake in northern Poland (54°15′9.522″N, 22°48′36.86″E). Animals were transported to the INC PAS by car in aerated travel containers. In the laboratory, the predators were kept by species in aquaria with 52 L of dechlorinated and aerated tap water in the same cabinet at a constant temperature of 20°C. The densities of predators in aquaria were based on the basal metabolic rate equations obtained for perch from [50] and for crayfish from [51]. To keep total metabolic rates balanced, the biomass of crayfish was made two times higher than the biomass of fish. After weighing, we kept two specimens of signal crayfish (wet mass 100 g) and two specimens of perch (wet mass 41.5 g) per experimental aquarium. We fed all predators frozen chironomid larvae (IT-Ichtyo Trophic, Stare Polichno, Poland) every second day (the larvae were thawed prior to feeding) and earthworms (My Worms, Słupsk, Poland) once a week. Once a week, we changed 10 L of water in the predator aquaria. Perches were collected and housed with permission from the Local Ethical Committee (ref. 394/2020). Signal crayfish were collected with permission from the Regional Directorate for Environmental Protection in Białystok (ref. WPN.6205.21.2020.ML) and Nature Reserve Hańcza Lake and housed with permission from the Regional Directorate for Environmental Protection in Kraków (ref. OP-I.672.8.2020.MK1).

Response variables

Survival was measured as the number of larvae that survived until day 14 after hatching, and until the day after adult emergence (hereafter, until emergence). Development time was measured as the number of days between oviposition and hatching (egg development time), and between hatching and emergence (larval development time). We also expressed development times in degree days (DDs), as DD better reflect the fact that insect development is strongly dependent on temperature. We calculated degree days by adding the average number of temperature degrees during the egg or larval stages. The lower temperature threshold for measuring DDs was 10°C (the winter period in both temperature treatment groups was excluded when calculating DDs). We used this threshold because the minimum temperature of larval development in Odonata ranges between 8-12ºC [52]. Adult mass at emergence was measured as wet mass one day after emergence. Animals were weighed to the nearest 0.1 mg using an electronic balance (Radwag® AS.62. R2 Plus).

Unfortunately, during the course of the experiment, we accidentally lost 27 individuals (Table S3). Because of this, some treatment groups ended up with low sample sizes and were not considered in the analysis. Sample size per combination of temperature, predator cue treatment, and population ranged between 14 and 50 eggs, and between 3 and 22 larvae. There were two larval groups with a sample size of one individual (SC group from Dąbski population under warming, and CC group from Płaszowski pond under current temperature).

Statistical analyses

For statistical analyses, we used R version 4.0.3 (R Core Team, 2013; RStudio Team, 2015). To test for survival, a generalized model (glm function) with binomial error distribution was used. For analysing egg and larval development times in days and in DDs, and body mass, separate linear

models (lm function) were used for each of the traits. Predator cues (control, perch and signal crayfish), temperature (current and warming), population (Dąbski and Płaszowski), and sex were added as explanatory variables. Because the sex could not be determined at the start of the experiment, sex could not be added to the survival analysis. Initially, we ran full models that included all main effects and interaction terms. Subsequently, the interaction terms with p > 0.05 were removed from the final models, and sex, if not significant, was also removed from the final models to increase the power of the test.

Results

Our statistical analyses showed significant main effects of predator cues, temperature, population, and sex, and in some cases two and three-way interaction effects between these variables on the life history of *I. elegans*.

Egg development time

In general, predator cues increased, and warming decreased the egg development time expressed in days. Eggs took the longest time for their development when exposed to signal crayfish cues, and the shortest time in the control treatment without predator cues. Across all predator cue treatment groups, eggs from Dąbski pond took more time for their development than eggs from Płaszowski pond, but only under the current temperature treatment (predator cue × temperature × population interaction, Figure 2A, Table 1).

Table 1. Effects of predator cues, temperature, population, and their interactions on egg development time in days and in degree-days (DDs) in *I. elegans*. Significant p-values are in bold.

**Predictor**	**df**	**Chisq**	**p-value**
**Egg development time in days**			
Egg predator cue	2	347.889	**< 0.001**
Temperature	1	1629.014	< **0.001**

Population	1	10.064	**0.002**
Predator cue x temperature	2	71.196	< **0.001**
Predator cue x population	2	30.483	< **0.001**
Temperature x population	1	5.348	**0.021**
Predator cue x temperature x population	2	12.669	< **0.001**
**Egg development time in DDs**			
Egg predator cue	2	314.139	< **0.001**
Temperature	1	600.066	< **0.001**
Population	1	9.156	**0.003**
Predator cue x temperature	2	67.980	< **0.001**
Predator cue x population	2	27.427	**< 0.001**
Temperature x population	1	4.865	**0.028**
Predator cue x temperature x population	2	17.442	**< 0.001**

Predator cues increased and warming decreased the egg development time expressed in degree-days (DDs). Eggs exposed to the signal crayfish cues required the most DDs, and eggs from the control group required the least DDs. Across all predator treatment groups, larvae from the Dąbski population needed more DDs than those from the Płaszowski population, but only under the current temperature treatment (Figure 2B, Table 1).

Figure 2. Effects of predator cues and temperature (current and 4°C warming) on the egg development time in days (A) and degree-days (B) in *I. elegans* from two populations (Dąbski and Płaszowski). Error bars show 95% CI. Sample sizes per group are provided in (A). Abbreviations: C – control; P- perch cues; S- signal crayfish cues.

Larval survival

Predator cues negatively affected larval survival until day 14 after hatching, but only under warming (interaction predator cue × temperature, Figure 3A, Table 2). Predator cues negatively affected survival in all groups, except for the chronic signal crayfish treatment at Płaszowski population (interaction predator cue × population Figure 3A, Table 2). Both acute and chronic perch exposure in the Płaszowski population and acute perch exposure in the Dąbski population caused the lowest survival rate (interaction predator cue × population, Figure 3A, Table 2).

Table 2. Effects of predator cues, temperature, population on larval survival (14 days after hatching and until emergence), development times in days and in degree-days (DDs), and mass in *I. elegans* (sex included). Significant p-values are in bold.

**Predictor**	**df**	**Chisq**	**p-value**
**Larval survival 14 days after hatching**			
Predator cue	4	6.210	0.184
Temperature	1	1.057	0.304
Population	1	1.057	0.304
Predator cue x temperature	4	18.724	**< 0.001**
Predator cue x population	4	14.634	**0.006**
**Larval survival until emergence**			
Predator cue	4	19.257	**0.001**
Temperature	1	7.638	**0.006**
Population	1	4.628	**0.031**
Predator cue x temperature	4	15.229	**0.004**
Predator cue x population	4	16.760	**0.002**
**Larval development time in days**			
Predator cue	4	2.225	0.069

Temperature	1	319.196	**< 0.001**
Population	1	5.414	**0.021**
**Larval development time in DDs**			
Predator cue	4	1.945	0.105
Temperature	1	141.886	**< 0.001**
Population	1	5.632	**0.019**
**Mass**			
Predator cue	4	3.031	**0.020**
Temperature	1	67.440	**< 0.001**
Population	1	4.412	**0.037**
Sex	1	10.334	**0.001**

Larval survival until emergence was positively affected by predator cues and warming. There was a significant population effect with the Płaszowski population showing higher survival (~50%) than the Dąbski (~40%) population (Figure 3B, Table 2). Warming positively affected survival only in the control, acute, and chronic perch cue treatments (interaction predator cue × temperature, Figure 3B, Table 2). Both acute and chronic exposure to perch and signal crayfish cues increased survival, but only in the Płaszowski population (interaction predator cue × population), whereas in the Dąbski population, only individuals under chronic perch cue exposure showed increased survival (interaction predator cue × population; Figure 3B, Table 2).

Figure 3. Effects of predator cues and temperature (current and 4°C warming) on larval survival after 14 days (A) and until emergence (B) in *I. elegans* from two populations (Dąbski and Płaszowski). Error bars show 95% CI. Abbreviations: CC - control (egg) and control (larva) group; PC - perch (egg) and control (larva) group; PP - perch (egg) and perch (larva) group; SC - signal

crayfish (egg) and control (larva) group; SS - signal crayfish (egg) and signal crayfish (larva) group.

Larval development time

There was a trend ($p = 0.076$) that chronic exposure to perch and signal crayfish cues increased larval development time expressed in days. Warming decreased larval development time. Larvae of the Dąbski population required more days than those of the Płaszowski population for larval development. None of the interaction terms were significant (Figure 4A, Table 2).

Predator cues showed no significant effects on larval development expressed in DDs. Warming decreased the DDs. Larvae from the Dąbski population expressed more DDs in the larval stage than those from the Płaszowski pond. None of the interaction terms were significant (Figure 4B, Table 2).

Figure 4. Effects of predator cues and temperature (current and warming) on the larval development time in days (A) and degree-days (B) in *I. elegans* from two populations (Dąbski and Płaszowski). Error bars show 95% CI. The sample size per group is provided in (A). Abbreviations as in Figure 3.

Mass at emergence

Predator cues significantly increased mass at emergence, and this pattern was mainly driven by the acute signal crayfish cue (SC) exposure (note that this group in the Dąbski population was excluded from the analysis due to low sample size), indicating a positive carry-over effect of signal crayfish cues from the egg until adult emergence (Figure 5, Table 2). After adding development time as a covariate, the increase in mass under predator stress was still significant (Chisq = 5.072, df = 4, $p = 0.001$), indicating that prolonged development under predator cues could not completely explain the mass increase. Warming increased mass at emergence. Individuals from the Dąbski

population were heavier than those from the Płaszowski population. Females were heavier than males. No interaction terms were significant (Figure 5, Table 2).

Figure 5. Effects of predator cues and temperature (current and warming) on the estimated mass at emergence in *I. elegans* from two populations (Dąbski and Płaszowski). Error bars show 95% CI. Sample size per group is provided. Abbreviations as in Figure 3.

Discussion

Our results revealed population-specific life history responses to NCEs from native and invasive alien predators and to warming at a microgeographic scale. Another important finding was that NCEs strongly affected egg development time and also carried over to larval and adult stages. These results point out that a full life cycle approach is needed as even when a stressor is experienced during a brief initial egg stage it can shape adult fitness traits through carry-over effects.

Larval survival decreased under predation risk, but only under warming conditions and until 14 days after hatching. Similar to the current results, perch cues decreased larval survival of *I. elegans* during the first two weeks after hatching [17]. In contrast to previous studies on damselflies [17,53] larval survival up to emergence under predation risk increased, but only in individuals from the Płaszowski pond. We do not have a clear explanation for the increased survival until emergence under predator stress. Nevertheless, it is plausible that the elevated survival until emergence could be attributed to acclimation to stress; individuals that managed to survive under predator stress during the egg and early larval stages may have subsequently exhibited reduced responses after continued exposure to the same predator cues throughout the subsequent larval stages and up to emergence.

Increased survival until emergence under warming and acute perch cue treatments (during the egg stage only in the Płaszowski population) and chronic perch cue treatment (during both egg and larval stages in the Dąbski population) may be explained by the fact that increased temperature shortens the exposure period to predation cues. Also, the positive warming effect indicated that the rearing temperature during a simulated peak of the growth season is below the optimal temperature for survival rate in *I. elegans*, as indicated in previous studies on this damselfly [13,54]. In general, this result promotes a positive carry-over effect as larvae exposed to perch cues only in the egg stage (acute exposition) showed higher survival from egg to adult emergence.

Separate analyses of NCE during the egg and larval stages showed that the difference in development time under predation pressure was driven mainly by egg responses to predator cues. This matches the idea that the immobile egg stage is the most sensitive phase to predation and further indicates that the negative predator-induced carry-over effect from the egg through the larval stage up to adult emergence decreases with exposure duration during the larval stage. Delayed development under predation pressure might be an effect of physiological stress in prey. Such stress could lead to re-allocation of energy to costly defence mechanisms instead of maintaining a fast development rate [12]. The effects of predator cues on development have been shown in previous studies. For example, *I. elegans* showed a longer egg development time in response to signal crayfish cues, which supports our results, but no response toward perch cues [38], which is in contrast to current results. Larvae briefly exposed to fish cues during initial larval instars decreased their growth rate in later instars [55]. Another study on confamiliar species showed either decreased (*Coenagrion pulchellum*) or increased (*Enallagma cyathigerum*) egg development times under predation risk [55]. These contrasting responses to predator cues might be due to population-specific experience with different predator species in nature and different

rearing methods, e.g. constant vs. changing temperatures and photoperiods. Conspecific populations from different habitats may respond differently when exposed to the same stressor. For example, in two independent studies on *I. elegans* eggs, one showed a significant effect of perch cues on egg development time [56], while the other study revealed no effect of perch cues on egg development time [38]. NCE on prey development might vary considerably between prey developmental stages, populations, and species, likely due to different defence systems used by prey [57].

A stronger delay in development under predator stress was found when individuals were exposed to the invasive crayfish cues than the native perch cues in the egg stage, and under chronic (egg and larval stages) than acute (egg stage only) exposition. The presence of an intense reaction to kairomones from the invasive crayfish might be explained by damselfly exposure to phylogenetically related crayfish species [58] that have been recorded in the two studied ponds (Table S4). A stronger response under chronic exposure to predator cues could result from weak or no physiological reconfiguration in the prey, in which prey reduces the costs of physiological adjustments under risk conditions [59]. Although the pattern of responses to predator cues during the larval stage was similar across the two populations and temperatures, the response of the egg stage was not consistent between both populations. Specifically, eggs from the Dąbski pond took more time for their development under predator stress than eggs from the Płaszowski pond, but only at the current temperature. A higher sensitivity to predator cues by eggs from the Dąbski pond than from the Płaszowski pond might be a consequence of damselfly experience with, respectively, multiple and single crayfish species in nature (Table S4). This suggestion is based on earlier studies showing a stronger effect of multiple predator species on prey responses to predation risk [26,60–62]. For example, *Culex restuans* mosquitoes that experienced a high level of background

predation risk based on pond-population predation history, showed increased wriggling activity in response to predation cues [26]. Warming reduced the NCE on egg development time likely due to faster development hence shorter exposure time under warming temperature as shown in the study of [63] and/or faster biodegradation of predator cues at the higher temperature, which was also found in previous case studies [64]. Such reduced NCEs under warming also removed the difference between populations in the egg response to predator cues. In summary, the results imply that egg and larval stages show different sensitivity to predator stress in terms of development rate and that warming may decrease this sensitivity and lead to similar responses to native and invasive predators.

When we expressed development time in terms of thermal units, i.e. degree-days (DDs), required for development, we noted qualitatively the same results as for estimates of development time expressed in days (compare Figures 2A and 4A vs. Figures 2B and 4B). Because the experimental simulation of seasonal changes in temperature was equally experienced by all reared individuals, may suggest that patterns in development time in days and DDs reflected thermal phenotypic evolution in the two local populations. However, maternal effects may partially have contributed to phenotypic differences between populations [65]. Studies on the contribution of maternal effects in damselfly offspring life history traits indicated that a considerable proportion of variance in egg, but not larval, traits was driven by maternal effects [66,67].

Damselflies from both populations showed an increased mass at emergence when exposed to predator cues, and this result was mainly driven by an acute egg exposure to signal crayfish, i.e. a positive carry-over effect from the egg to adult emergence. A higher mass under predation stress was not just a side-effect of prolonged development as the pattern remained the same after correcting mass by development time. The stronger effect of crayfish than perch cues suggests that

damselfly eggs are more prone to stress caused by predation risk from crayfish than from perch. Juvenile and mature signal crayfish are omnivorous and feed on substrates into which *I. elegans* eggs might be oviposited, with faster crayfish growth if the substrate contains invertebrates [68]. In comparison, juvenile and mature perch feed on mobile prey such as zooplankton, invertebrates, and fish [69], hence the fish is less likely to prey upon damselfly eggs.

In both temperature treatments, damselflies from the warmer Płaszowski pond expressed a shorter development time in days and DDs until emergence than individuals from the colder Dąbski pond, and this regardless of the predator cue treatment. Such thermal differentiation in development might reflect cogradient variation where both genes and warmer environment direct population towards fastest development [70], a pattern less often reported in ectotherms [71,72]. In our case this may reflect up-regulation of the expression of genes regulating development in the population from the warmer pond, and down-regulation of these genes in the population from the colder pond. Such pattern of up-regulated genes in individuals reared under elevated temperature was shown in *I. elegans* larvae from several other populations in southern Poland [73]. Furthermore, as a warming climate leads to an extension of the growth season, this can lead to an increased number of generations per growth season, i.e. from univoltine to bivoltine [33]. Note that the thermal differentiation between populations appeared despite a strong homogenization effect of gene flow at a microgeographic scale, as was indicated in previous population genetic studies on mobile ectotherms [25,74].

Experimental warming shortened the development time of individuals from both populations and increased mass at emergence. In ectotherms, increasing temperatures elevate the development and growth rates which shape the body size, here reflected as body mass. Ectotherms that follow the reverse temperature size rule (TSR) phenomenon, as found in our case, are expected

to be more temperature sensitive for biomass accumulation rather than for ontogenetic development [75]. Similar results in which body size increased with increased temperature were documented in some odonates [76], amphibians [77] and fishes [78,79]. An increased size under warming could have a positive impact on fecundity; usually, larger females have higher potential fecundity than smaller ones [80], but see [81]. In contrast, other studies on *I. elegans* showed no or the opposite pattern where elevated temperatures decreased mass at emergence [13,29,56,82]. In these cases, larvae were exposed to strong intraspecific competition which might cause the discrepancy in the results. Previous and current findings suggest that it is not straightforward to predict future changes in ectotherms' size under warming condition using simple experimental studies.

When comparing between populations, the adaptive response to xxx what? xxx with a faster completion of pre-emergence development in the Płaszowski compared to the Dąbski population was traded off with a lower mass at emergence. Therefore, fixed population-specific differences in mass with lighter individuals from the warmer than the colder pond opposed the plastic thermal responses expressed during the experiment by both populations. This suggests that short-term ecological responses to warming might result in an increased mass, whereas long-term evolutionary responses in mass gain might be constrained by cogradient thermal variation in an associated trait, development rate. As mentioned, maternal effects that were not analysed here could also play a role in shaping mass. This makes it challenging to conclude to what degree the direction of thermal response in mass is driven by plasticity, genes, or maternal effects.

To summarize, our results revealed the presence of population- and life stage-specific responses in life-history traits to NCEs from native and invasive predators and warming. These population-specific responses to ecological stressors were recorded at a microgeographic scale,

suggesting a phenotypic signal of adaptation despite a likely strong gene flow. The interacting effects of warming and predator stress on egg and early instars larval fitness traits revealed a strong synergy between these stressors, yet, these interactions weakened or disappeared during subsequent larval development up to emergence, resulting in additive stressor effects. Stage-specific responses to (combined) stressors highlight that a full life cycle approach is required to predict adult fitness through carry-over effects of stressors experienced in egg and larval stages.

References:

1. Kaunisto S, Ferguson L V., Sinclair BJ. Can we predict the effects of multiple stressors on insects in a changing climate? Curr Opin Insect Sci. 2016;17: 55–61. doi:10.1016/j.cois.2016.07.001
2. Birk S, Chapman D, Carvalho L, Spears BM, Andersen HE, Argillier C, et al. Impacts of multiple stressors on freshwater biota across spatial scales and ecosystems. Nat Ecol Evol. 2020;4: 1060–1068. doi:10.1038/s41559-020-1216-4
3. Simmons BI, Blyth PSA, Blanchard JL, Clegg T, Delmas E, Garnier A, et al. Refocusing multiple stressor research around the targets and scales of ecological impacts. Nat Ecol Evol. 2021;5: 1478–1489. doi:10.1038/s41559-021-01547-4
4. Orr JA, Vinebrooke RD, Jackson MC, Kroeker KJ, Kordas RL, Mantyka-Pringle C, et al. Towards a unified study of multiple stressors: Divisions and common goals across research disciplines. Proc R Soc B Biol Sci. 2020;287. doi:10.1098/rspb.2020.0421
5. Martin CW. Naïve prey exhibit reduced antipredator behavior and survivorship. PeerJ. 2014;2014. doi:10.7717/peerj.665
6. Steindler LA, Blumstein DT, West R, Moseby KE, Letnic M. Exposure to a novel predator induces visual predator recognition by naïve prey. Behav Ecol Sociobiol. 2020;74. doi:10.1007/s00265-020-02884-3
7. Ferrari MCO, Crane AL, Brown GE, Chivers DP. Getting ready for invasions: Can background level of risk predict the ability of naïve prey to survive novel predators? Sci Rep. 2015;5: 1–7. doi:10.1038/srep08309
8. Sih A, Bolnick DI, Luttbeg B, Orrock JL, Peacor SD, Pintor LM, et al. Predator-prey naïveté, antipredator behavior, and the ecology of predator invasions. Oikos. 2010;119: 610–621. doi:10.1111/j.1600-0706.2009.18039.x
9. Cox JG, Lima SL. Naiveté and an aquatic-terrestrial dichotomy in the effects of introduced predators. Trends Ecol Evol. 2006;21: 674–680. doi:10.1016/j.tree.2006.07.011
10. Neven LG. Physiological responses of insects to heat. Postharvest Biol Technol. 2000;21: 103–111. doi:10.1016/S0925-5214(00)00169-1
11. Kingsolver JG, Arthur Woods H, Buckley LB, Potter KA, MacLean HJ, Higgins JK. Complex life cycles and the responses of insects to climate change. Integr Comp Biol. 2011;51: 719–732. doi:10.1093/icb/icr015

12. Hawlena D, Schmitz OJ. Physiological stress as a fundamental mechanism linking predation to ecosystem functioning. Am Nat. 2010;176: 537–556. doi:10.1086/656495
13. Raczyński M, Stoks R, Johansson F, Bartoń K, Sniegula S. Phenological Shifts in a Warming World Affect Physiology and Life History in a Damselfly. Insects. 2022;13. doi:10.3390/insects13070622
14. Alexander JM, Diez JM, Hart SP, Levine JM. When Climate Reshuffles Competitors: A Call for Experimental Macroecology. Trends Ecol Evol. 2016;31: 831–841. doi:10.1016/j.tree.2016.08.003
15. Carter SK, Rudolf VHW. Shifts in phenological mean and synchrony interact to shape competitive outcomes. Ecology. 2019;100: e02826. doi:10.1002/ecy.2826
16. Bailey LD, van de Pol M. Tackling extremes: Challenges for ecological and evolutionary research on extreme climatic events. J Anim Ecol. 2016;85: 85–96. doi:10.1111/1365-2656.12451
17. Sniegula S, Raczyński M, Golab MJ, Johansson F. Effects of predator cues carry over from egg and larval stage to adult life-history traits in a damselfly. Freshw Sci. 2020;39: 804–811. doi:10.1086/711374
18. Stoks R, Crdoba-Aguilar A. Evolutionary ecology of Odonata: A complex life cycle perspective. Annu Rev Entomol. 2012;57: 249–265. doi:10.1146/annurev-ento-120710-100557
19. Ower GD, Juliano SA. The demographic and life-history costs of fear: Trait-mediated effects of threat of predation on Aedes triseriatus. Ecol Evol. 2019;9: 3794–3806. doi:10.1002/ece3.5003
20. Paterson RA, Pritchard DW, Dick JTA, Alexander ME, Hatcher MJ, Dunn AM. Predator cue studies reveal strong trait-mediated effects in communities despite variation in experimental designs. Anim Behav. 2013;86: 1301–1313. doi:10.1016/j.anbehav.2013.09.036
21. Ituarte RB, Vázquez MG, González-Sagrario M de los Á, Spivak ED. Carryover effects of predation risk on postembryonic life-history stages in a freshwater shrimp. Zoology. 2014;117: 139–145. doi:10.1016/j.zool.2013.09.004
22. Stoks R, De Block M, Slos S, Van Doorslaer W, Rolff J. Time constraints mediate predator-induced plasticity in immune function, condition, and life history. Ecology. 2006;87: 809–815. doi:10.1890/0012-9658(2006)87[809:TCMPPI]2.0.CO;2
23. Tüzün N, Op de Beeck L, Brans KI, Janssens L, Stoks R. Microgeographic differentiation in thermal performance curves between rural and urban populations of an aquatic insect. Evol Appl. 2017;10: 1067–1075. doi:10.1111/eva.12512
24. Chen B, Kang L. Insect population differentiation in response to environmental thermal stress. Prog Nat Sci. 2005;15: 289–296. doi:10.1080/10020070512331342130
25. Orizaola G, Laurila A. Microgeographic variation in temperature-induced plasticity in an isolated amphibian metapopulation. Evol Ecol. 2009;23: 979–991. doi:10.1007/s10682-008-9285-x
26. Ferrari MCO, Messier F, Chivers DP. Variable predation risk and the dynamic nature of mosquito antipredator responses to chemical alarm cues. Chemoecology. 2007;17: 223–229. doi:10.1007/s00049-007-0380-1
27. Stevenson RJ, Sabater S. Understanding effects of global change on river ecosystems: Science to support policy in a changing world. Hydrobiologia. 2010;657: 3–18. doi:10.1007/s10750-010-0392-7

28. Hassall C, Thompson DJ. The effects of environmental warming on odonata: A review. Int J Odonatol. 2008;11: 131–153. doi:10.1080/13887890.2008.9748319
29. Palomar G, Wos G, Stoks R, Sniegula S. Latitude-specific urbanization effects on life history traits in the damselflyIschnura elegans. bioRxiv. 2023. Available: https://doi.org/10.1101/2023.02.27.530143
30. Chown SL, Gaston KJ. Macrophysiology for a changing world. Proc R Soc B Biol Sci. 2008;275: 1469–1478. doi:10.1098/rspb.2008.0137
31. Janssens L, Van Dievel M, Stoks R. Warming reinforces nonconsumptive predator effects on prey growth, physiology, and body stoichiometry. Ecology. 2015;96: 3270–3280. doi:10.1890/15-0030.1
32. Dijkstra K. Field Guide to the Dragonflies of Britain and Europe . Second edition . 2020.
33. Corbet PS, Suhling F, Soendgerath D. Voltinism of odonata: A review. Int J Odonatol. 2006;9: 1–44. doi:10.1080/13887890.2006.9748261
34. Corbet PS. Dragonflies: Behavior and Ecology of Odonata. 1999.
35. Van De Meutter F, De Meester L, Stoks R. Water turbidity affects predator-prey interactions in a fish-damselfly system. Oecologia. 2005;144: 327–336. doi:10.1007/s00442-005-0050-3
36. Schaffner AK, Anholt BR. Influence of predator presence and prey density on behavior and growth of damselfly larvae (Ischnura elegans) (Odonata: Zygoptera). J Insect Behav. 1998;11: 793–809. doi:10.1023/a:1020803925186
37. Gall M Le, Chaput-Bardy A, Husté A. Context-dependent local movements of the blue-tailed damselfly, Ischnura elegans: effects of pond characteristics and the landscape matrix. J Insect Conserv. 2017;21: 243–256. doi:10.1007/s10841-017-9971-5
38. Antoł A, Sniegula S. Damselfly eggs alter their development rate in the presence of an invasive alien cue but not a native predator cue. Ecol Evol. 2021;11: 9361–9369. doi:10.1002/ece3.7729
39. Sánchez-Guillén RA, Hansson B, Wellenreuther M, Svensson EI, Cordero-Rivera A. The influence of stochastic and selective forces in the population divergence of female colour polymorphism in damselflies of the genus Ischnura. Heredity (Edinb). 2011;107: 513–522. doi:10.1038/hdy.2011.36
40. Froese R, Pauly D. FishBase. World Wide Web electronic publication. 2014.
41. Dobrzycka-Krahel A, Skóra ME, Raczyński M, Szaniawska A. The Signal Crayfish Pacifastacus leniusculus — Distribution and Invasion in the Southern Baltic Coastal River. Polish J Ecol. 2017;65: 445–452. doi:10.3161/15052249PJE2017.65.3.011
42. Sniegula S, Golab MJ, Drobniak SM, Johansson F. The genetic variance but not the genetic covariance of life-history traits changes towards the north in a time-constrained insect. J Evol Biol. 2018;31: 853–865. doi:10.1111/jeb.13269
43. IPCC. IPCC press release AR6. Clim Chang 2013 - Phys Sci Basis. 2021; 1–6.
44. Mebane CA, Schmidt TS, Miller JL, Balistrieri LS. Bioaccumulation and Toxicity of Cadmium, Copper, Nickel, and Zinc and Their Mixtures to Aquatic Insect Communities. Environ Toxicol Chem. 2020;39: 812–833. doi:10.1002/etc.4663
45. Johansson F, Watts PC, Sniegula S, Berger D. Natural selection mediated by seasonal time constraints increases the alignment between evolvability and developmental plasticity. Evolution (N Y). 2021;75: 464–475. doi:10.1111/evo.14147
46. Lake Model FLake. 2009. FLake online. http://www.flake. igb-berlin.de/index.shtml Latimer,

47. Dinh Van K, Janssens L, Debecker S, Stoks R. Temperature- and latitude-specific individual growth rates shape the vulnerability of damselfly larvae to a widespread pesticide. J Appl Ecol. 2014;51: 919–928. doi:10.1111/1365-2664.12269
48. Van Buskirk J, Krügel A, Kunz J, Miss F, Stamm A. The Rate of Degradation of Chemical Cues Indicating Predation Risk: An Experiment and Review. Ethology. 2014;120: 942–949. doi:10.1111/eth.12266
49. De Block M, Stoks R. Adaptive sex-specific life history plasticity to temperature and photoperiod in a damselfly. J Evol Biol. 2003;16: 986–995. doi:10.1046/j.1420-9101.2003.00581.x
50. Enders EC, Boisclair D, Boily P, Magnan P. Effect of body mass and water temperature on the standard metabolic rate of juvenile yellow perch, Perca flavescens (Mitchill). Environ Biol Fishes. 2006;76: 399–407. doi:10.1007/s10641-006-9045-0
51. Wheatly MG. Standard Rate of O2 Uptake and Body Size in the Crayfish Pacifastacus Leniusculus (Dana, 1852) (Decapoda: Astacidae): Intra- Versus Interspecific Relations in Crustaceans. J Crustac Biol. 1989;9: 212–216. doi:10.1163/193724089X00034
52. Suhling F, Suhling I, Richter O. Temperature response of growth of larval dragonflies – an overview. Int J Odonatol. 2015;18: 15–30. doi:10.1080/13887890.2015.1009392
53. Fontana-Bria L, Selfa J, Tur C, Frago E. Early exposure to predation risk carries over metamorphosis in two distantly related freshwater insects. Ecol Entomol. 2017;42: 255–262. doi:10.1111/een.12382
54. Van Dievel M, Tüzün N, Stoks R. Latitude-associated evolution and drivers of thermal response curves in body stoichiometry. J Anim Ecol. 2019;88: 1961–1972. doi:10.1111/1365-2656.13088
55. Sniegula S, Nsanzimana J d. A, Johansson F. Predation risk affects egg mortality and carry over effects in the larval stages in damselflies. Freshw Biol. 2019;64: 778–786. doi:10.1111/fwb.13261
56. Raczyński M, Stoks R, Sniegula S. Warming and predation risk only weakly shape size-mediated priority effects in a cannibalistic damselfly. Sci Rep. 2022;12: 1–12. doi:10.1038/s41598-022-22110-6
57. Sugiura S. Predators as drivers of insect defenses. Entomol Sci. 2020;23: 316–337. doi:10.1111/ens.12423
58. Anton A, Geraldi NR, Ricciardi A, Dick JTA. Global determinants of prey naiveté to exotic predators. Proc R Soc B Biol Sci. 2020;287. doi:10.1098/rspb.2019.2978
59. Jermacz Ł, Kletkiewicz H, Nowakowska A, Dzierżyńska-Białończyk A, Klimiuk M, Kobak J. Continuity of chronic predation risk determines changes in prey physiology. Sci Rep. 2020;10: 1–8. doi:10.1038/s41598-020-64000-9
60. Martin CW, Fodrie FJ, Heck KL, Mattila J. Differential habitat use and antipredator response of juvenile roach (Rutilus rutilus) to olfactory and visual cues from multiple predators. Oecologia. 2010;162: 893–902. doi:10.1007/s00442-010-1564-x
61. Ueshima E, Yusa Y. Antipredator behaviour in response to single or combined predator cues in the apple snail Pomacea canaliculata. J Molluscan Stud. 2015;81: 51–57. doi:10.1093/mollus/eyu057
62. Walzer A, Schausberger P. Integration of multiple cues allows threat-sensitive anti-intraguild predator responses in predatory mites. Behaviour. 2013;150: 115–132. doi:10.1163/1568539X-00003040
63. Touchon JC, Warkentin KM. Thermally contingent plasticity: Temperature alters

expression of predator-induced colour and morphology in a Neotropical treefrog tadpole. J Anim Ecol. 2011;80: 79–88. doi:10.1111/j.1365-2656.2010.01765.x
64. Chivers DP, Dixson DL, White JR, Mccormick MI, Ferrari MCO. Degradation of chemical alarm cues and assessment of risk throughout the day. Ecol Evol. 2013;3: 3925–3934. doi:10.1002/ece3.760
65. Moore MP, Whiteman HH, Martin RA. A mother's legacy: the strength of maternal effects in animal populations. Ecol Lett. 2019;22: 1620–1628. doi:10.1111/ele.13351
66. Sniegula S, Golab MJ, Drobniak SM, Johansson F. Seasonal time constraints reduce genetic variation in life-history traits along a latitudinal gradient. J Anim Ecol. 2016;85: 187–198. doi:10.1111/1365-2656.12442
67. Shama LNS, Campero-Paz M, Wegner KM, De Block M, Stoks R. Latitudinal and voltinism compensation shape thermal reaction norms for growth rate. Mol Ecol. 2011;20: 2929–2941. doi:10.1111/j.1365-294X.2011.05156.x
68. Bondar CA, Bottriell K, Zeron K, Richardson JS. Does trophic position of the omnivorous signal crayfish (Pacifastacus leniusculus) in a stream food web vary with life history stage or density? Can J Fish Aquat Sci. 2005;62: 2632–2639. doi:10.1139/f05-167
69. Freyhof, J. & Kottelat M. Perca fluviatilis. The IUCN Red List of Threatened Species. Version 2014.3. Http://WwwIucnredlistOrg/Details/16580/0. 2008;8235. Available: http://www.iucnredlist.org/details/16580/0
70. Conover D 0., Schultz ET. Phenotypic similarity and the evolutionary significance of countergradient variation. Trends Ecol Evol. 1995;10: 248–252. doi:10.1016/S0169-5347(00)89081-3
71. Tüzün N, Stoks R. Evolution of geographic variation in thermal performance curves in the face of climate change and implications for biotic interactions. Curr Opin Insect Sci. 2018;29: 78–84. doi:10.1016/j.cois.2018.07.004
72. Conover DO, Duffy TA, Hice LA. The covariance between genetic and environmental influences across ecological gradients: Reassessing the evolutionary significance of countergradient and cogradient variation. Ann N Y Acad Sci. 2009;1168: 100–129. doi:10.1111/j.1749-6632.2009.04575.x
73. Wos G, Palomar G, Marszałek M, Babik W, Sniegula S. The effect of temperature and invasive alien predator on genetic and phenotypic variation in the damselfly Ischnura elegans: cross-latitude comparison. Front Zool. 2023;20: 1–14. doi:10.1186/s12983-023-00494-z
74. Richardson JL, Urban MC, Bolnick DI, Skelly DK. Microgeographic adaptation and the spatial scale of evolution. Trends Ecol Evol. 2014;29: 165–176. doi:10.1016/j.tree.2014.01.002
75. Zuo W, Moses ME, West GB, Hou C, Brown JH. A general model for effects of temperature on ectotherm ontogenetic growth and development. Proc R Soc B Biol Sci. 2012;279: 1840–1846. doi:10.1098/rspb.2011.2000
76. Sniegula S, Golab MJ, Johansson F. A large-scale latitudinal pattern of life-history traits in a strictly univoltine damselfly. Ecol Entomol. 2016;41: 459–472. doi:10.1111/een.12314
77. Olalla-Tárraga MÁ, Rodríguez MÁ. Energy and interspecific body size patterns of amphibian faunas in Europe and North America: Anurans follow Bergmann's rule, urodeles its converse. Glob Ecol Biogeogr. 2007;16: 606–617. doi:10.1111/j.1466-8238.2007.00309.x

78. Horne CR, Hirst AG, Atkinson D. Temperature-size responses match latitudinal-size clines in arthropods, revealing critical differences between aquatic and terrestrial species. Ecol Lett. 2015;18: 327–335. doi:https://doi.org/10.1111/ele.12413
79. Forster J, Hirst AG, Atkinson D. Warming-induced reductions in body size are greater in aquatic than terrestrial species. Proc Natl Acad Sci. 2012;109: 19310–19314. doi:10.1073/pnas.1210460109
80. Sokolovska N, Rowe L, Johansson F. Fitness and body size in mature odonates. Ecol Entomol. 2000;25: 239–248. doi:10.1046/j.1365-2311.2000.00251.x
81. Fincke OM, Hadrys H. Unpredictable offspring survivorship in the damselfly, Megaloprepus coerulatus, shapes parental behavior, constrains sexual selection, and challenges traditional fitness estimates. Evolution. 2001;55: 762–772. doi:10.1554/0014-3820(2001)055[0762:uositd]2.0.co;2
82. Verheyen J, Stoks R. Temperature variation makes an ectotherm more sensitive to global warming unless thermal evolution occurs. J Anim Ecol. 2019;88: 624–636. doi:10.1111/1365-2656.12946

 Click here to access/download;Figure;Figures.docx

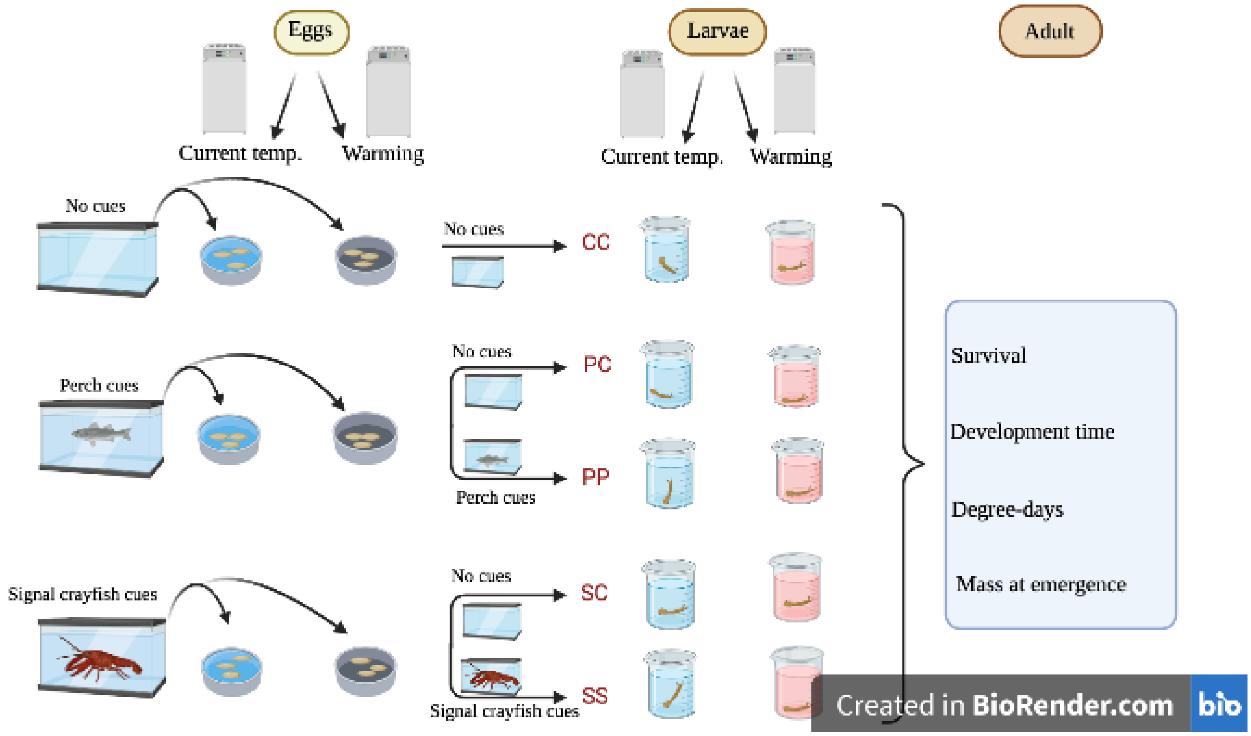

Figure 1. Experimental design. *Ischnura elegans* eggs were assigned to groups receiving no predator cues (control), native European perch cues (perch), or invasive alien signal crayfish cues (signal crayfish). At hatching, the egg control group continued as larval control group (CC). The egg perch cue group was divided into larval control (PC) and larval perch cue (PP) groups. The egg signal crayfish cue group was divided into larval control (SC) and larval signal crayfish cue (SS) groups.

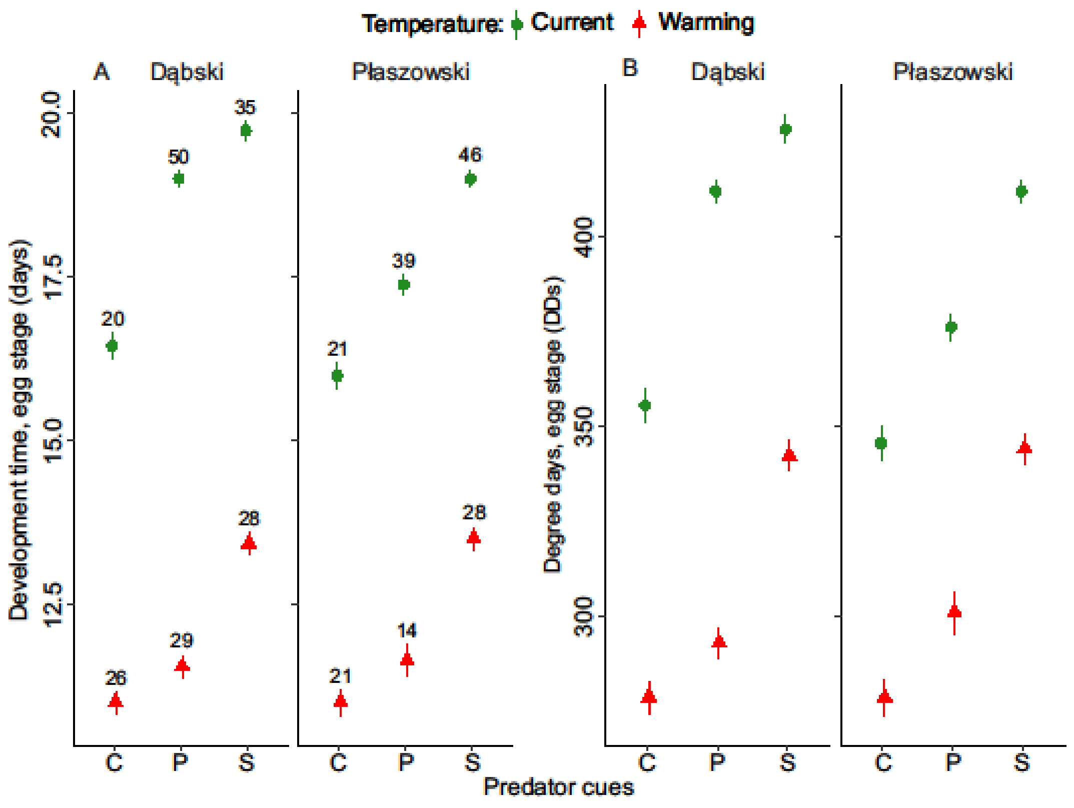

Figure 2. Effects of predator cues and temperature (current and warming) on the egg development time in days (A) and degree-days (B) in *I. elegans* from two populations (Dąbski and Płaszowski). Error bars show 95% CI. Sample sizes per group are provided in (A). Abbreviations: C – control; P- perch; S- signal crayfish cues.

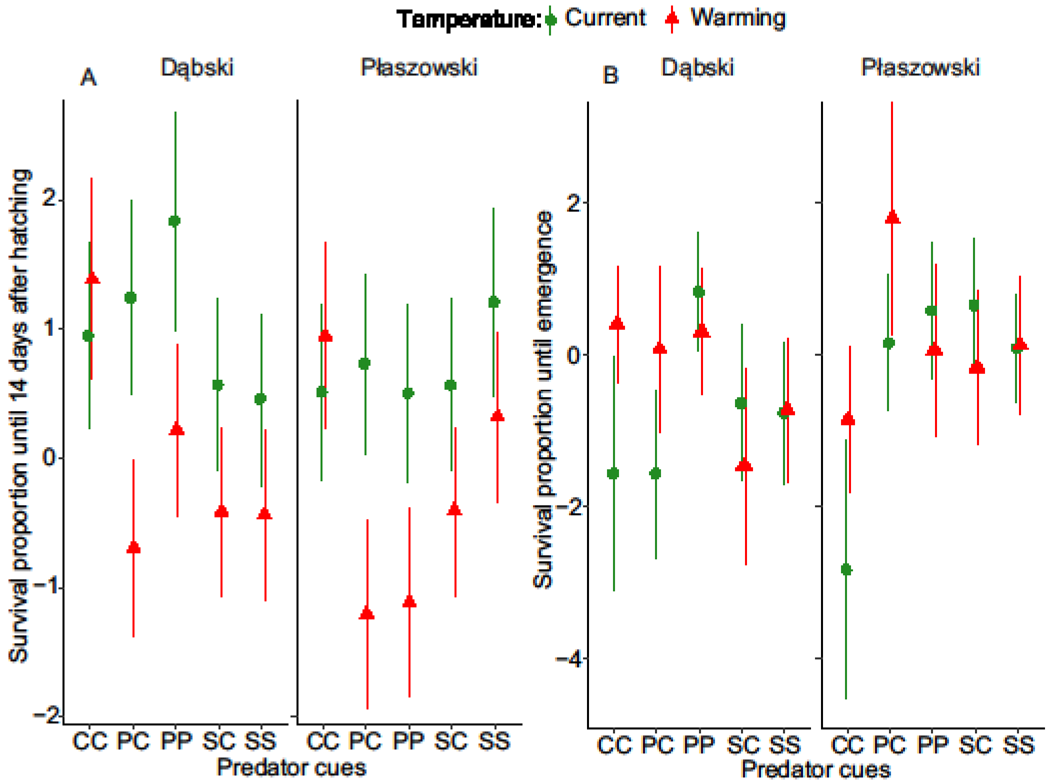

Figure 3. Effects of predator cues and temperature (current and warming) on larval survival after 14 days (A) and until emergence (B) in *I. elegans* from two populations (Dąbski and Płaszowski). Error bars show 95% CI. Abbreviations: CC - control (egg) and control (larva) group; PC - perch (egg) and control (larva) group; PP - perch (egg) and perch (larva) group; SC - signal crayfish (egg) and control (larva) group; SS - signal crayfish (egg) and signal crayfish (larva) group.

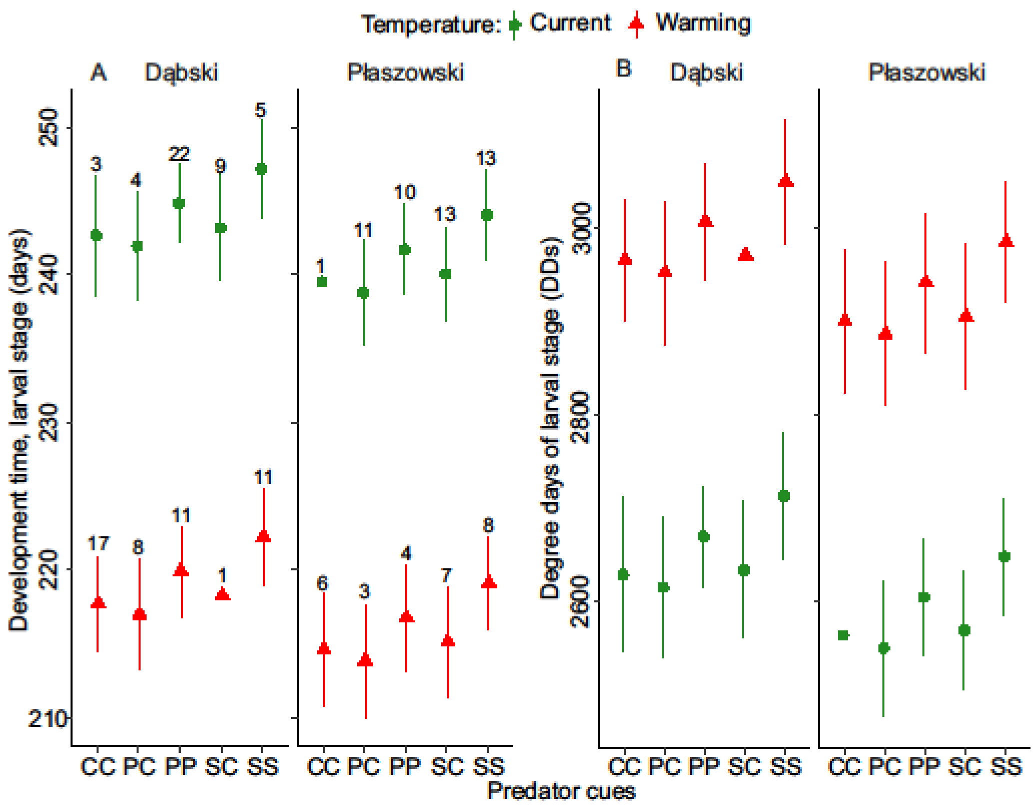

Figure 4. Effects of predator cues and temperature (current and warming) on the larval development time in days (A) and degree-days (B) in *I. elegans* from two populations (Dąbski and Płaszowski). Error bars show 95% CI. The sample size per group is provided in (A). Abbreviations as in Figure 3.

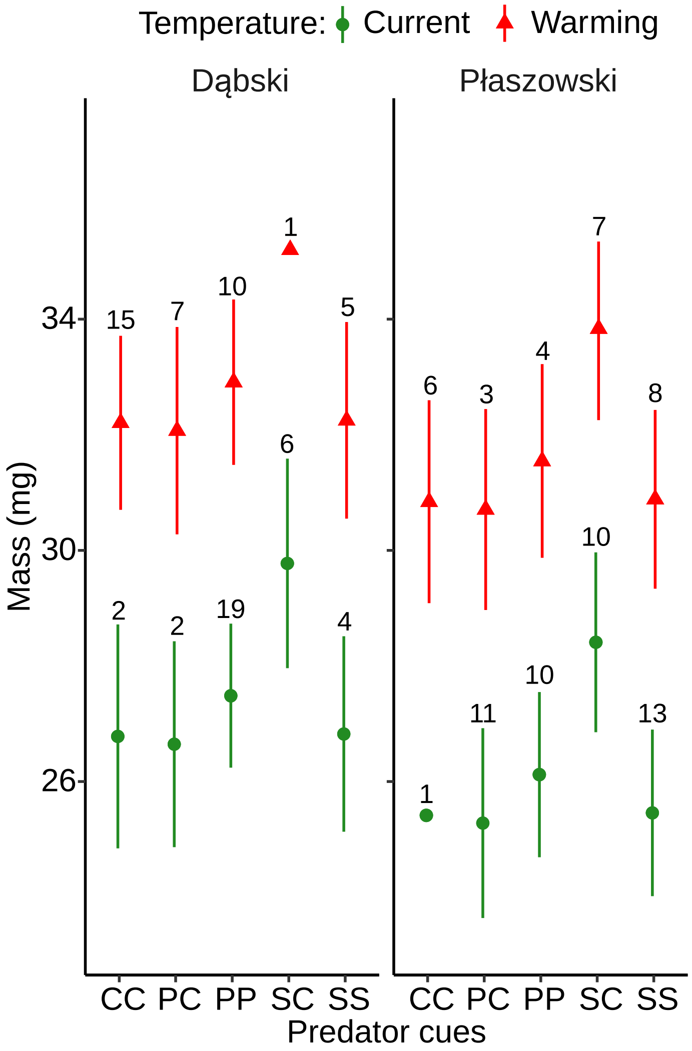

Figure 5. Effects of predator cues and temperature (current and warming) on the estimated mass at emergence in *I. elegans* from two populations (Dąbski and Płaszowski). Error bars show 95% CI. Sample size per group is provided. Abbreviations as in Figure 3.

Click here to access/download
Supporting Information
Supplementary material_szs_NR.docx
