# Supplementary material for: Microgeographic differentiation in thermal and antipredator responses and their carry-over effects across life stages in a damselfly
Source: PLoS One. 2024 Feb 23;19(2):e0295707. doi: 10.1371/journal.pone.0295707 (PMC10889876; doi:10.1371/journal.pone.0295707)
Supplement: Attachment — Submitted filename: Reviewer comments.docx [file pone.0295707.s010.docx]

Manuscripts Number: PONE-D-23-24816

Reviewer’s comment

The manuscript is well written and provides detail insight on the possible combined impact of global warming and the invasion of non-native predator species on the egg and larval behavior of the very common damselfly. This experimental work gives us new understanding about the overall prey population behaviors and also specific adaptive behavior at different life stages of prey species. Knowing the importance of the organism in the trophic structure and energy transfer and the information on how climate induced changes may lead to a cascading effect (top-down or bottom-up) is the need of hour. I would especially like to mention that, this research has adopted an experimental approach to address the combined effects of temperature rise and predation (native and non-native) effects on the life cycle of prey population. Though the experimental findings are difficult to acclimatize into real world environment, a setup of multiple test combinations have answered many questions and based on this observation the authors were able to demonstrate that, the ecologically important damselfly *Ischnura elegans* exhibits adaptive resilience to warming and predator stress.

I believe under ever-changing climate and ever-increasing human pressure on the world global ecosystem, this manuscript will fall in the line of global efforts of ecosystem conservation and future prediction model. This work also provided a promising finding which is quite opposite to the prevailing notion on the adverse impact due to the climate change which must be supported and substantiated later. Therefore, I recommend the manuscript has a potential for publication in **PLOSE ONE**. However, few of minor corrections from my side have been highlighted, corrected or deleted in the PDF file and request the authors to kindly incorporate the same as long as those are empirical. Since I am not a native of English speaking but still, I gave a try to correct few.
